# Dosage and safety of transcranial magnetic stimulation and transcranial direct current stimulation for managing patients with chronic musculoskeletal pain: a scoping review protocol

**DOI:** 10.1136/bmjopen-2025-103925

**Published:** 2025-09-09

**Authors:** Liming Jiang, Yifan Wang, Yuwu Ding, Kun Yang, Zengqiao Zhang, Ramakrishnan Mani, Jiayi Xia, Bitao Ma, Sizhong Wang

**Affiliations:** 1 Department of Rehabilitation, Seventh People’s Hospital of Shanghai University of Traditional Chinese Medicine, Shanghai, China; 2 School of Rehabilitation Medicine, Shanghai University of Traditional Chinese Medicine, Shanghai, China; 3 School of Physiotherapy, University of Otago, Dunedin, New Zealand; 4 Clinical Research Center, The Second Rehabilitation Hospital of Shanghai, Shanghai, China; 5 Department of Traditional Chinese Medicine, Xinhua Hospital Affiliated to Shanghai Jiao Tong University School of Medicine, Shanghai, China; 6 School of Exercise and Health, Shanghai University of Sport, Shanghai, China; 7 Department of Health Sciences, Brunel University of London, London, UK

**Keywords:** Chronic Pain, Musculoskeletal disorders, Transcranial Magnetic Stimulation

## Abstract

**Abstract:**

**Introduction:**

Although emerging evidence supports the short-term efficacy of transcranial magnetic stimulation (TMS), including repetitive TMS (rTMS) and theta-burst transcranial magnetic stimulation (TBS-TMS), and transcranial direct current stimulation (tDCS) for managing patients with chronic musculoskeletal pain (CMP), their clinical utility in managing CMP remains inconclusive. This uncertainty may arise from methodological limitations, including heterogeneity in treatment parameters such as stimulation targets and dosages. Additionally, safety profiles for these non-invasive brain stimulation interventions in patients with CMP remain insufficiently reported, with limited data on adverse events, cumulative risks and long-term safety outcomes. Hence, in this scoping review protocol, we aim to systematically (1) identify and map the stimulation targets and dosages of TMS and tDCS used in previous studies to treat patients with CMP; (2) summarise the rationale for using the stimulation targets and dosages of TMS and tDCS; and (3) summarise the reports on the safety of TMS and tDCS in managing patients with CMP, including whether safety was reported and how it was described.

**Methods and analysis:**

We will adopt Arksey and O’Malley’s methodological framework and report findings according to the Preferred Reporting Items for Systematic reviews and Meta-Analyses extension for Scoping Reviews. Two authors will independently screen articles and extract data from PubMed, Scopus, Web of Science and Embase, including publications from inception to 1 August 2025. Discrepancies regarding study inclusion will be resolved through consultation with a third author. We will synthesise results using descriptive statistical methods.

**Ethics and dissemination:**

This scoping review does not require ethical approval. The findings will be disseminated through presentation at national or international conferences and peer-reviewed publication.

**Trial registration details:**

Open Science Framework (Registration DOI: https://doi.org/10.17605/OSF.IO/8HFBZ).

**Strengths and limitations of this study**

- This review followed Arksey and O’Malley’s six-stage framework and adhered to Preferred Reporting Items for Systematic reviews and Meta-Analyses extension for Scoping Reviews guidelines, ensuring transparency and reproducibility in the scoping review process.
- Consultations with physiotherapists during the design stage provided valuable real-world clinical insights, enhancing the relevance and applicability of the review.
- We will include only studies published in English, which may result in language bias and limit the generalisability of the review findings.

## Introduction

 Chronic musculoskeletal pain (CMP) is pain that originates from muscles, bones, joints, ligaments, tendons or related structures and persists for more than 3 months.^1^ The multifaceted nature of CMP poses challenges in its management, as traditional pharmacological approaches often yield limited long-term benefits and may be associated with adverse effects.^2^ Conservative approaches, including lifestyle modifications, physiotherapy and psychological interventions, have demonstrated potential in alleviating symptoms. However, they frequently fail to provide complete pain relief.^2 3^

In recent years, non-invasive brain stimulation has emerged as a promising intervention for chronic pain, including CMP.^4 5^ This technique has the potential for addressing the underlying neurophysiological mechanisms of chronic pain by modulating cortical excitability and neural networks involved in pain processing.^6^ Among the various non-invasive brain stimulation techniques, transcranial magnetic stimulation (TMS), including repetitive TMS (rTMS) and theta-burst transcranial magnetic stimulation (TBS-TMS), and transcranial direct current stimulation (tDCS) have emerged as the most widely used interventions.^7 8^

The theoretical rationale for using brain stimulation in pain management is supported by neurophysiological models such as the Bayesian brain model.^9^ This model conceptualises pain as an imbalance between two ascending (lateral and medial) pathways and a descending pathway.^9^ The lateral pain pathway processes the sensory-discriminative aspects of pain (eg, painfulness) via the somatosensory cortex. The medial pain pathway processes the emotional-affective dimension (eg, suffering) and involves regions such as the rostral dorsal anterior cingulate cortex and anterior insula. In contrast, the descending pain modulatory system, including the pregenual anterior cingulate cortex and brainstem, helps regulate and suppress incoming pain signals. Modulating these pathways through non-invasive brain stimulations could provide new therapeutic opportunities for patients with CMP.^10^

Despite a growing body of research on TMS and tDCS, some guidelines and systematic reviews indicate that the effectiveness and safety of non-invasive brain stimulation for treating CMP remain inconclusive.^811^,^13^ This uncertainty may arise from inconsistencies in stimulation targets and dosage parameters across studies.^14^ Both TMS and tDCS have been applied to a variety of cortical targets, including primary motor cortex (M1), primary somatosensory cortex (S1), dorsal anterior cingulate cortex (dACC) and dorsolateral prefrontal cortex (DLPFC).^14^,^17^ However, variability in stimulation targets across studies limits our ability to draw definitive conclusions about which brain regions should be prioritised in clinical application. To date, no comprehensive synthesis has mapped how different brain stimulation techniques target specific cortical areas in the treatment of CMP, nor how these targets align with the Bayesian brain model.^9^

Similarly, the dosage of simulation varies across studies.^18^,^22^ The dosage of TMS is related not only to intensity, duration, number and frequency of therapeutic pulses, and treatment interval, but also to the coil geometry, pulse waveform, intensity, frequency and number of pulses delivered.^19 23^ The dosage of tDCS is related to current intensity, waveform and electrode size.^7^ These variations in stimulation targets and dosages may contribute to the heterogeneity of findings in TMS and tDCS research. Currently, clinical decision-making regarding stimulation parameters often relies on empirical knowledge rather than robust evidence.^7^

Safety remains another critical concern for the application of TMS and tDCS, as both techniques directly influence the central nervous system.^7^ The guidelines highlighted that, in studies that clearly report side effects, both real and sham stimulations have short-term and mild side effects, such as skin irritation, nausea and headache.^724^,^26^ However, there have been rare cases of epileptic seizures following real rTMS treatment.^7^ Although existing tDCS studies have not found serious adverse effects, the conclusions are limited to short-term treatment results.^27^ Furthermore, the safety reports of TMS and tDCS are not comprehensive, so the safety of these techniques needs further exploration.

Therefore, this review aims to systematically (1) identify and map stimulation targets and dosages of TMS and tDCS used in previous studies for the treatment of patients with CMP; (2) summarise the rationale for using specific stimulation dosages; and (3) summarise whether and how the safety of TMS and tDCS in patients with CMP was reported.

## Methods and analysis

### Patient and public involvement

Patients and public were not involved.

#### Design

We will report this review according to the Preferred Reporting Items for Systematic reviews and Meta-Analyses extension for Scoping Reviews (PRISMA-ScR) guidelines.^28^ We will follow the methodological framework established by Arksey and O’Malley,^29^ which includes six stages: (1) defining the research question; (2) identifying relevant studies; (3) study selection; (4) plotting and presenting data; (5) collating, summarising and reporting results; and (6) optional consultation with relevant stakeholders. We registered this scoping review in the Open Science Framework.

##### Phase 1: Identify research questions

This phase involved iterative discussions among the research team and stakeholder consultations with two physiotherapists (YD and KY) with clinical or research experience in managing patients with CMP through rTMS and tDCS. Based on discussions, we formulated the following research questions:

1. Which stimulation positions of TMS and tDCS have previous studies targeted?
2. What dosages of TMS and tDCS have previous studies used?
3. Do the authors provide rationale for using a specific dosage of TMS and tDCS?
4. Do previous studies report the safety of TMS and tDCS? If so, how have they reported the safety outcomes of TMS and tDCS?

##### Phase 2: Identification of relevant studies

###### Search strategy

We developed and finalised the search strategy after iterative discussions and preliminary exploratory searches. The lead author (SW) conducted a preliminary pilot search across PubMed, Scopus, Web of Science and Embase to identify relevant articles and review existing search strategies. Building on this, the lead author (SW) developed a complete search strategy by identifying key terms contained in the titles and abstracts of relevant articles. Given the broad spectrum of conditions classified under CMP, this review does not limit inclusion to any specific diagnosis. Instead, we adopted the general term ‘pain’ to capture a wide range of musculoskeletal pain conditions. This review specifically focuses on brain stimulation interventions involving rTMS, TBS-TMS or theta-burst stimulation (TBS), and tDCS. Accordingly, the search strategy includes the full terms and abbreviations for these modalities. Since the objective of these interventions is to improve pain and function, no additional conceptual restrictions were applied. The search strategy will be tailored to the specific requirements of each database. Furthermore, reference lists of included studies will be examined to identify any other relevant studies. The lead author (SW) will conduct the final search on 1 August 2025, using predefined keywords and subject headings (table 1) in the aforementioned databases.

**Table 1:** Keywords used in the literature search

Concept	Keywords
1	Pain
2	Repetitive transcranial magnetic stimulation, rTMS, transcranial direct current stimulation, tDCS, theta-burst transcranial magnetic stimulation, theta-burst stimulation, or TBS

### Phase 3: Study selection

#### Inclusion and exclusion criteria

##### Study population

We will include studies that recruited adults diagnosed with CMP, which is defined as pain lasting for more than 3 months due to persistent inflammation or structural changes in the musculoskeletal tissue (including muscles, bones, joints and/or connective tissue),^1^ such as osteoarthritis, fibromyalgia, myofascial pain syndrome, temporomandibular joint disorder, temporomandibular joint pain, neck pain, shoulder pain, rotator cuff tendinopathy, subacromial pain syndrome, back pain, patellofemoral pain syndrome, chronic ankle instability, ankle sprain, etc. To maintain conceptual clarity and a more homogeneous sample, we will exclude postoperative and viral CMP to avoid confounding the analysis of brain stimulation effects on musculoskeletal pain networks. These conditions involve distinct pathophysiological mechanisms, such as surgical trauma and immune or viral neuropathic processes, that differ from those seen in non-specific or degenerative CMP.

##### Concept

In this scoping review, we will include only studies that investigate brain stimulation interventions using TMS (including rTMS, TBS-TMS, and TBS), tDCS or both. To be eligible for inclusion, studies must report at least one of the following parameters: (1) stimulation targets, such as M1, DLPFC, dACC and S1; (2) stimulation dosages—for rTMS, the dosage includes the coil type, stimulation frequency, stimulation intensity, number of pulses, interval time and duration of treatment; for TBS, the dosage includes the coil type, stimulation pattern (intermittent TBS/ continuous TBS), burst parameters, stimulation intensity, total pulses per session and treatment duration; for tDCS, the dosage includes the anode position, cathode position, electrode size, current intensity, stimulation time, rise and fall time, and duration of treatment; and (3) safety assessment to evaluate adverse effects or risks associated with the interventions.

##### Context

We will include studies conducted in any setting where the intervention is administered or supervised by clinicians, such as a physiotherapist.

##### Evidence source type

We will include only full-text studies published in English. To comprehensively map the existing evidence, we will consider a broad range of study designs, including randomised controlled trials (pilot, feasibility or full-scale), crossover trials, case–control studies and observational studies. We will also include protocols if the corresponding full-text results are not yet published. We will exclude review articles, clinical practice guidelines, expert opinions, books or book chapters, editorials, letters to the editor, and conference abstracts or proceedings.

##### Screening

Duplicate articles will first be removed using EndNote software. Two independent authors will then screen the titles and abstracts before conducting a full-text review. Any disagreements regarding study inclusion will be resolved through discussion, and if necessary, a third author will be consulted to reach a consensus.

### Phase 4: Data extraction

Two authors will independently extract data from all included studies, with any disagreements resolved through discussion. If consensus is not achieved, a third author will be consulted. For studies with incomplete data, the corresponding authors will be contacted via email to request missing details. Data extraction will be conducted using three preliminary extraction forms (tables2^5^), which will be modified if necessary.

**Table 2:** Characteristics of included studies

**Author, year, and country**	**Study design**	**Participants** **(sample size and CMP condition)**	**Intervention**	**Comparator**	**Outcome measures**

CMP, chronic musculoskeletal pain.

**Table 3:** Stimulation targets and dosages of repetitive transcranial magnetic stimulation

**Study**	**Stimulation target**	**Coil type**	**Dosage**					**Adverse events**
			**Frequency (Hz)**	**Intensity (MT, %)**	**Total number of pulses**	**Interstimulation interval**	**Treatment session(s)**	

**Table 4:** Stimulation targets and dosages of theta-burst transcranial magnetic stimulation

**Study**	**Stimulation target**	**Coil type**	**Dosage**					**Adverse events**
			**Stimulation pattern**	**Burst parameters**	**Intensity**	**Total pulse per session**	**Treatment session(s)**	

**Table 5:** Stimulation targets and dosages of transcranial direct current stimulation

**Study**	**Stimulation target**		**Electrode size**	**Dosage**				**Adverse events**
	**Anode position**	**Cathode position**		**Current intensity**	**Stimulation time**	**Rise time and fall time**	**Treatment session(s)**	

We will extract general characteristics of the included studies in table 2. The general characteristics include: (1) study characteristics (authors, year of publication and study location); (2) study design; (3) details of participants (sample size and CMP condition); (4) interventions and comparators (such as TMS or tDCS); and (5) outcome measures (key parameters assessed). We will also extract stimulation target (such as M1, DLPFC, dACC and S1), coil type of rTMS and dosage parameters for rTMS and TBS in tables3  4, respectively. Similarly, we will extract stimulation target, electrode size of tDCS and dosage parameters for tDCS in table 5. For both TMS and tDCS, we will extract detailed dosage information, such as stimulation intensity, frequency, duration of each session, total number of sessions and overall treatment schedule. Additionally, if a study provides a rationale or justification for the selected dosage, we will extract this information along with any reported safety considerations. These justifications may be based on prior studies, neurophysiological mechanisms, dose-response relationships or theoretical models.

### Phase 5: Collating, summarising and reporting results

The results of the literature search and process of study screening will be presented using a PRISMA-ScR flow chart. We will synthesise results through descriptive analysis and report findings using tables, graphs or visual maps.

Given the heterogeneity of conditions included under CMP (eg, fibromyalgia, rotator cuff tendinopathy, chronic ankle instability), we will organise and present findings using subgroupings based on anatomical region (eg, spine, upper limb, lower limb) and/or pain characteristics (eg, widespread vs localised pain). This stratification aims to improve the conceptual coherence of the review and allow for more meaningful interpretation of the evidence.

Regarding the stimulation target, we will map stimulation targets through a two-level analysis: first categorising by anatomical brain regions (such as M1, DLPFC, ACC and S1), then classifying by functional networks (triple network model: default mode, salience and central executive networks). This approach will help us determine whether specific pain phenotypes correlate with particular network modulations. We will also assess how studies connect their stimulation targets to theoretical models, like predictive coding or Bayesian brain hypotheses. This evaluation will identify where current evidence supports these frameworks and where gaps remain in our theoretical understanding.

To manage the variability in stimulation parameters, we will implement a structured synthesis strategy. Studies will be categorised by key intervention characteristics, including stimulation modality (TMS, tDCS or combined), stimulation frequency (eg, low-frequency vs high-frequency rTMS; anodal vs cathodal tDCS) and dosage parameters (eg, intensity, session duration, number of sessions). Although no universally accepted framework exists for interpreting dosage rationale in non-invasive brain stimulation for CMP, we will summarise the rationale authors provide for selecting specific parameters. This systematic approach will support thematic organisation of the findings, facilitate identification of trends across clinical contexts and reveal inconsistencies or underexplored areas in current intervention design.

### Phase 6: Consultation

This review serves as a foundational component of a broader research initiative aimed at advancing treatments for CMP. To identify any missing or relevant studies not included in the review, stakeholder consultations will be conducted in two phases. During the protocol development stage, we engaged two experienced practising physiotherapists with clinical expertise in managing CMP. These stakeholders were selected based on their practical experience with brain stimulation interventions and their involvement in multidisciplinary pain management teams. Consultations were conducted through structured interviews. Their input informed the refinement of the research questions, inclusion criteria and the selection of stimulation targets and dosage parameters, ensuring that the review remained clinically grounded from the start. In the second phase, we will share preliminary findings with two physiotherapy experts to obtain their interpretations based on clinical expertise. Their insights will help contextualise the results and refine the review’s conclusions, ensuring alignment with real-world therapeutic practices. This iterative engagement will enhance the review’s clinical relevance and strengthen its utility for guiding future research and treatment strategies for CMP.

## Discussion

This scoping review aims to systematically identify and map the stimulation targets and stimulation dosages, and safety profiles of TMS and tDCS used in the treatment of patients with CMP. By examining the diversity of stimulation targets, this review will contribute to a clearer understanding of how these techniques align with emerging theoretical models of pain, such as the Bayesian brain model and the triple network hypothesis.^9 10^ This theory-informed mapping strategy may uncover patterns in how brain stimulation interventions are designed and justified, as well as identify underexplored or novel targets with potential therapeutic relevance. Furthermore, by synthesising stimulation parameters and their reported rationale, the review may support the development of more standardised and mechanism-driven treatment protocols, moving beyond empirically driven practices to promote evidence-based clinical decision-making.

Safety remains a fundamental concern, especially for interventions that directly affect brain function. While side effects of non-invasive brain stimulation are generally considered mild and transient, rare but serious adverse events such as seizures highlight the need for comprehensive and consistent safety monitoring.^30^ By mapping how safety is reported across existing studies, this review will identify gaps in adverse event reporting and suggest areas for improvement in future clinical trials.

However, several limitations should be acknowledged. As a scoping review, this study will not assess the quality or risk of bias of the included studies, which may limit the interpretability of our findings, particularly with respect to the reliability of reported outcomes and the safety of the interventions. Readers should interpret the results with caution, especially in areas where evidence is limited or derived from studies with small sample sizes or unclear methodology. Additionally, due to the anticipated heterogeneity in study designs, patient populations and intervention protocols, synthesis of findings may be primarily descriptive rather than quantitative. Another limitation is the limited scope of stakeholder involvement. Consultation was restricted to two physiotherapists who are also co-authors, which, while helpful in shaping the research questions and data extraction framework, may not fully capture the perspectives of broader stakeholder groups. The absence of independent clinical experts, patient representatives or policy-makers may limit the external validity and practical relevance of the review. Despite these limitations, the findings of this review will provide important insights into the current landscape of non-invasive brain stimulation interventions for CMP. The results may inform future systematic reviews, clinical trials and guideline development aimed at optimising the use of brain stimulation techniques for managing chronic pain. Ultimately, this review will support researchers and clinicians in developing more targeted, effective and safer neurostimulation strategies for patients with CMP.

### Ethics and dissemination

This protocol does not require ethical approval. Systematic synthesis of stimulation parameters (targets and dosages) reported in previously published studies using TMS and/or tDCS for managing patients with CMP will inform the selection of optimal protocols in future research. The results will be disseminated through presentation at national or international conferences and peer-reviewed publications.
