# Supplementary material for: Dosage and safety of transcranial magnetic stimulation and transcranial direct current stimulation for managing patients with chronic musculoskeletal pain: a scoping review protocol
Source: BMJ Open. 2025 Sep 9;15(9):e103925. doi: 10.1136/bmjopen-2025-103925 (PMC12421619; doi:10.1136/bmjopen-2025-103925)
Supplement: Supplementary file 1 [file bmjopen-2025-103925.reviewer_comments.pdf]

PEER REVIEW HISTORY

BMJ Open publishes all reviews undertaken for accepted manuscripts. Reviewers are asked to complete a checklist review form and are provided with free text boxes to elaborate on their assessment. These free text comments are reproduced below.

ARTICLE DETAILS

Title (Provisional)

Dosage and safety of transcranial magnetic stimulation and transcranial direct current stimulation for managing patients with chronic musculoskeletal pain: a scoping review protocol

Authors

Jiang, Li-ming; Wang, Yifan; Ding, Yuwu; Yang, Kun; Zengqiao, Zhang; Mani, Ramakrishnan; Xia, Jiayi; Ma, Bitao; Wang, Sizhong

---

VERSION 1 - REVIEW

**Reviewer**	**1**
**Name**	**Tedeschi, Roberto**
**Affiliation**	**University of Bologna, Bologna, Italy.**
**Date**	**17-Jun-2025**
**COI**	**None**

While the manuscript provides a well-structured and timely protocol for a scoping review on rTMS and tDCS in chronic musculoskeletal pain (CMP), several critical issues should be addressed to improve methodological rigour and conceptual clarity:

Mismatch between Scoping Review Purpose and PICOS Framework

Although the authors clearly identify this as a scoping review, the application of the PICOS criteria is more typical of a systematic review. This introduces ambiguity, as scoping reviews are intended to explore rather than evaluate effectiveness.

Restriction to RCTs Only

Including only randomised controlled trials (RCTs) is overly restrictive for a scoping review, which is designed to map the breadth of evidence. This choice may inadvertently exclude relevant studies—particularly observational or feasibility studies—that report on stimulation targets, dosage, and safety.

Lack of Methodological Quality Appraisal

While omitting risk-of-bias assessment is acceptable in some scoping reviews, the authors should acknowledge how this limitation may affect the interpretability of the findings, especially concerning safety outcomes.

Overly Broad Definition of CMP

The inclusion criteria cover heterogeneous conditions (e.g., fibromyalgia, rotator cuff tendinopathy, and chronic ankle instability), which may compromise the conceptual coherence of the review. Subgrouping or stratification strategies should be considered.

Exclusion of Postoperative and Viral CMP

The rationale for excluding chronic musculoskeletal pain caused by surgery or viral inflammation is unclear and unjustified. These are clinically relevant conditions where non-invasive brain stimulation could be highly applicable.

Unclear Plan for Assessing Dosage Rationales

It remains ambiguous how the rationale for stimulation dosage will be extracted or evaluated. Will any framework or existing guidelines be used to interpret these justifications?

Superficial Integration of Theoretical Models

Although the Bayesian brain model and triple network hypothesis are briefly mentioned, there is no clear plan for how these will guide data synthesis or interpretation. Their relevance should be better integrated into the mapping strategy.

No Protocol Registration

The authors should consider registering the protocol (e.g., with PROSPERO or OSF) and providing the registration number to enhance transparency and reduce duplication.

Limited Stakeholder Involvement

Stakeholder consultation appears limited to two physiotherapists who are also co-authors. Broader input from independent clinical experts, patients, or policy-makers would improve the external validity of the review.

No Strategy to Manage Heterogeneity

The anticipated heterogeneity in stimulation parameters is not addressed. A pre-specified strategy to handle this (e.g., categorisation by cortical target, stimulation frequency, or duration) would strengthen the analytical framework.

Additional Recommendation

The authors should also consider citing recent and relevant literature in this field, including:

Transcranial direct current stimulation for chronic foot pain: A comprehensive review (10.1016/j.ensci.2024.100498)

Transcranial Direct Current Stimulation in the Treatment of Chronic Knee Pain: A Scoping Review (10.3390/app14167100)

VERSION 1 - AUTHOR RESPONSE

Reviewer: 1

Dr. Roberto Tedeschi, University of Bologna, Bologna, Italy.

Comments to the Author:

While the manuscript provides a well-structured and timely protocol for a scoping review on rTMS and tDCS in chronic musculoskeletal pain (CMP), several critical issues should be addressed to improve methodological rigour and conceptual clarity:

Response: Thank you for taking the time to review our manuscript and for your constructive comments. We improved the manuscript as per your comments point by point.

Mismatch between Scoping Review Purpose and PICOS Framework

Although the authors clearly identify this as a scoping review, the application of the PICOS criteria is more typical of a systematic review. This introduces ambiguity, as scoping reviews are intended to explore rather than evaluate effectiveness.

Response: Thank you for your insightful comment. We have revised the search strategy, inclusion and exclusion criteria, and Table 1 accordingly.

While the PICOS framework is traditionally used in systematic reviews to evaluate intervention effectiveness, we have selectively adapted components, specifically, Population, Intervention, and Outcome, to guide the development of our eligibility criteria and search strategy. This adaptation serves to enhance transparency and structure without implying an evaluative approach. Our scoping review remains exploratory in nature, consistent with its aim to map key concepts, stimulation parameters, and theoretical frameworks related to non-invasive brain stimulation in chronic musculoskeletal pain (CMP).

In line with the methodological guidance for scoping reviews, no formal comparison between interventions or outcome synthesis will be undertaken. To avoid potential ambiguity, we have refined both the search strategy and the inclusion/exclusion criteria, and we have updated Table 1 to better reflect the scope and objectives of the review.

The revised search strategy reads as “We developed and finalised the search strategy after iterative discussions and preliminary exploratory searches. The lead author (S.W.) conducted a preliminary pilot search across PubMed, Scopus, Web of Science, and Embase to identify relevant articles and review existing search strategies. Building on this, the lead author (S.W.) developed a complete search strategy by identifying key terms contained in the titles and abstracts of relevant articles. Given the broad spectrum of conditions classified under CMP, this review does not limit inclusion to any specific diagnosis. Instead, we adopted the general term “pain” to capture a wide range of musculoskeletal pain conditions. This review specifically focuses on brain stimulation interventions involving repetitive transcranial magnetic stimulation (rTMS), theta-burst transcranial magnetic stimulation or theta-burst stimulation (TBS), and transcranial direct current stimulation (tDCS). Accordingly, the search strategy includes the full terms and abbreviations for these modalities. Since the objective of these interventions is to improve pain and function, no additional conceptual restrictions were applied. The search strategy will be tailored to the specific requirements of each database. Furthermore, reference lists of included studies will be examined to identify any other relevant studies. The lead author (S.W.) will conduct the final search on 1 August 2025, utilising predefined keywords and subject headings (Table 1) in the aforementioned databases.”

The revised Study population section reads as “We will include studies that recruited adults diagnosed with CMP. The CMP is defined as pain lasting for more than three months due to persistent inflammation or structural changes in the musculoskeletal tissue (including muscles, bones, joints and/or connective tissue),1 such as osteoarthritis, fibromyalgia, myofascial pain syndrome, temporomandibular joint disorder, temporomandibular joint pain, neck pain, shoulder pain, rotator cuff tendinopathy, subacromial pain syndrome, back pain, patellofemoral pain syndrome, chronic ankle instability, ankle sprain, etc. To maintain conceptual clarity and a more homogeneous sample, we will exclude postoperative and viral CMP to avoid confounding the analysis of brain stimulation effects on musculoskeletal pain networks. These conditions involve distinct pathophysiological mechanisms, such as surgical trauma and immune or viral neuropathic processes, that differ from those seen in non-specific or degenerative CMP.”

The revised Table 1 is presented below:

Table 1. Keywords used in literature search

Concept	Keywords
1	Pain
2	Repetitive transcranial magnetic stimulation, rTMS, transcranial direct current stimulation, tDCS, theta-burst transcranial magnetic stimulation, theta-burst stimulation, or TBS

Restriction to RCTs Only

Including only randomised controlled trials (RCTs) is overly restrictive for a scoping review, which is designed to map the breadth of evidence. This choice may inadvertently exclude relevant studies—particularly observational or feasibility studies—that report on stimulation targets, dosage, and safety.

Response: Thank you for your constructive comment. We revised Evidence source type section as follows: "We will include only full-text studies published in English. To comprehensively map the existing evidence, we will consider a broad range of study designs, including randomized controlled trials (pilot, feasibility, or full-scale), crossover trials, case-control studies, and observational studies. We will also include protocols if the corresponding full-text results are not yet published. We will exclude review articles, clinical practice guidelines, expert opinions, books or book chapters, editorials, letters to the editor, and conference abstracts or proceedings."

Lack of Methodological Quality Appraisal

While omitting risk-of-bias assessment is acceptable in some scoping reviews, the authors should acknowledge how this limitation may affect the interpretability of the findings, especially concerning safety outcomes.

Response: Thank you for highlighting this important point. We have improved the limitation of omitting risk-of-bias assessment and the revised information reads as: "As a scoping review, this study will not assess the quality or risk of bias of the included studies, which may limit the interpretability of our findings, particularly with respect to the reliability of reported outcomes and the safety of the interventions. Readers should interpret the results with caution, especially in areas where evidence is limited or derived from studies with small sample sizes or unclear methodology.".

Overly Broad Definition of CMP

The inclusion criteria cover heterogeneous conditions (e.g., fibromyalgia, rotator cuff tendinopathy, and chronic ankle instability), which may compromise the conceptual coherence of the review. Subgrouping or stratification strategies should be considered.

Response: We appreciate the reviewer's comment on the broad definition of chronic musculoskeletal pain (CMP) in our inclusion criteria. Our intention was to capture the full scope of how brain stimulation techniques have been applied across a wide range of CMP conditions. However, we acknowledge that the inclusion of heterogeneous conditions may limit conceptual coherence and complicate interpretation.

The added information in Phase 5: Collating, summarizing, and reporting results section reads "Given the heterogeneity of conditions included under CMP (e.g., fibromyalgia, rotator cuff tendinopathy, chronic ankle instability), we will organise and present findings using subgroupings based on anatomical region (e.g., spine, upper limb, lower limb) and/or pain characteristics (e.g., widespread vs. localised pain). This stratification aims to improve the conceptual coherence of the review and allow for more meaningful interpretation of the evidence.".

Exclusion of Postoperative and Viral CMP

The rationale for excluding chronic musculoskeletal pain caused by surgery or viral inflammation is unclear and unjustified. These are clinically relevant conditions where non-invasive brain stimulation could be highly applicable.

Response: Thank you for your comment. We acknowledge that CMP following surgery or viral inflammation (e.g., post-COVID myalgia or post-surgical joint pain) is clinically relevant and may benefit from non-invasive brain stimulation (NIBS). However, we have chosen to exclude these conditions to maintain conceptual clarity and ensure a more homogeneous sample of CMP etiologies. Postoperative and viral CMP often involve distinct pathophysiological mechanisms, such as surgical tissue trauma, immune-mediated inflammation, or viral neuropathic components, which may differ from the mechanisms underlying non-specific or degenerative CMP (e.g., osteoarthritis, tendinopathy). Including these diverse origins could confound the interpretation of how brain stimulation targets pain-related brain networks in musculoskeletal conditions. We believe this narrower focus will enhance the coherence of our review findings and allow for more meaningful synthesis.

We added justification for excluding postoperative and Viral CMP and the added information reads as "To maintain conceptual clarity and a more homogeneous sample, we will exclude postoperative and viral CMP to avoid confounding the analysis of brain stimulation effects on musculoskeletal pain networks. These conditions involve distinct pathophysiological mechanisms, such as surgical trauma and immune or viral neuropathic processes, that differ from those seen in non-specific or degenerative CMP." in Study population section.

Unclear Plan for Assessing Dosage Rationales

It remains ambiguous how the rationale for stimulation dosage will be extracted or evaluated. Will any framework or existing guidelines be used to interpret these justifications?

Response: Thank you for your valuable comment. We have clarified our plan for extracting and interpreting stimulation dosage rationales. We added further information in Phase 4: Data Extraction section and the added information reads as "Additionally, if a study provides a rationale or justification for the selected dosage, we will extract this information along with any reported safety considerations. These justifications may be based on prior studies, neurophysiological mechanisms, dose-response relationships, or theoretical models.". We also added information to interpret rationale for dosage selection in Phase 5: Collating, summarising, and reporting results section and the added information reads as "Although no universally accepted framework exists for interpreting dosage rationale in non-invasive brain stimulation for CMP, we will summarise the rationale authors provide for selecting specific parameters. This systematic approach will support thematic organization of the findings, facilitate identification of trends across clinical contexts, and reveal inconsistencies or underexplored areas in current intervention design.".

Superficial Integration of Theoretical Models

Although the Bayesian brain model and triple network hypothesis are briefly mentioned, there is no clear plan for how these will guide data synthesis or interpretation. Their relevance should be better integrated into the mapping strategy.

Response: We appreciate the reviewer's comment and agree that a stronger integration of theoretical models is essential for framing and interpreting the findings.

We added more information on data synthesis and interpretation and the added information reads as "Regarding the stimulation target, we will map stimulation targets through a two-level analysis: first categorizing by anatomical brain regions (such as M1, DLPFC, ACC, and S1),

then classifying by functional networks (triple network model: default mode, salience, and central executive networks). This approach will help us determine whether specific pain phenotypes correlate with particular network modulations. We will also assess how studies connect their stimulation targets to theoretical models like predictive coding or Bayesian brain hypotheses. This evaluation will identify where current evidence supports these frameworks and where gaps remain in our theoretical understanding." in Phase 5: Collating, summarizing, and reporting results section.

We also revised the first paragraph of discussion section and the revised discussion reads as "This scoping review aims to fill this gap by systematically identifying and mapping the stimulation targets and stimulation dosages and safety profiles of TMS and tDCS used in the treatment of patients with CMP. By examining the diversity of stimulation targets, this review will contribute to a clearer understanding of how these techniques align with emerging theoretical models of pain, such as the Bayesian brain model and the triple network hypothesis.[8, 23] This theory-informed mapping strategy may uncover patterns in how brain stimulation interventions are designed and justified, as well as identify underexplored or novel targets with potential therapeutic relevance. Furthermore, by synthesizing stimulation parameters and their reported rationale, the review may support the development of more standardized and mechanism-driven treatment protocols, moving beyond empirically driven practices to promote evidence-based clinical decision-making.".

No Protocol Registration

The authors should consider registering the protocol (e.g., with PROSPERO or OSF) and providing the registration number to enhance transparency and reduce duplication.

Response: Thank you for your comment. We registered the scoping review protocol in OSF and the Registration DOI is: https://doi.org/10.17605/OSF.IO/8HFBZ .

Limited Stakeholder Involvement

Stakeholder consultation appears limited to two physiotherapists who are also co-authors.

Broader input from independent clinical experts, patients, or policy-makers would improve the external validity of the review.

Response: Thank you for your comment. We acknowledged the limitation of limited stakeholder involvement and the added information reads as "Another limitation is the limited scope of stakeholder involvement. Consultation was restricted to two physiotherapists who are also co-authors, which, while helpful in shaping the research questions and data extraction framework, may not fully capture the perspectives of broader stakeholder groups. The absence of independent clinical experts, patient representatives, or policy-makers may limit the external validity and practical relevance of the review.". We also added more information on consultation and the added information reads as "we engaged two experienced practising physiotherapists with clinical expertise in managing CMP. These stakeholders were selected based on their practical experience with brain stimulation interventions and their involvement in multidisciplinary pain management teams. Consultations were conducted through structured interviews. Their input informed the refinement of the research questions, inclusion criteria, and the selection of stimulation targets and dosage parameters, ensuring that the review remained clinically grounded from the start.".

No Strategy to Manage Heterogeneity

The anticipated heterogeneity in stimulation parameters is not addressed. A pre-specified strategy to handle this (e.g., categorisation by cortical target, stimulation frequency, or duration) would strengthen the analytical framework.

Response: Thank you for your constructive comment. We added strategy to handle anticipated heterogeneity in stimulation parameters.

The added information reads as "To manage the variability in stimulation parameters, we will implement a structured synthesis strategy. Studies will be categorized by key intervention characteristics, including stimulation modality (rTMS, tDCS, or combined), stimulation frequency (e.g., low- vs. high-frequency rTMS; anodal vs. cathodal tDCS), and dosage parameters (e.g., intensity, session duration, number of sessions). Where available, we will also extract and summarize the rationale authors provide for selecting specific parameters. This systematic approach will support thematic organization of the findings, facilitate identification of trends across clinical contexts, and reveal inconsistencies or underexplored areas in current intervention design." in Phase 5: Collating, summarizing, and reporting results section.

Additional Recommendation

The authors should also consider citing recent and relevant literature in this field, including: Transcranial direct current stimulation for chronic foot pain: A comprehensive review (10.1016/j.ensci.2024.100498)
Transcranial Direct Current Stimulation in the Treatment of Chronic Knee Pain: A Scoping Review (10.3390/app14167100)

Response: Thank you for your helpful suggestion. We have now cited the two recent and relevant reviews as recommended.

VERSION 2 - REVIEW

**Reviewer**	**1**
**Name**	**Tedeschi, Roberto**
**Affiliation**	**University of Bologna, Bologna, Italy.**
**Date**	**02-Aug-2025**
**COI**	

Thnak you
